# How innovation funding leads enterprises to engage in research and development: Small and medium enterprises’ perspective

**DOI:** 10.1371/journal.pone.0289166

**Published:** 2023-07-25

**Authors:** Hui Sun, Xiaocong Yang, Xuan Tang, Fei Peng

**Affiliations:** 1 School of Economics and Statistics, Guangzhou University, Guangzhou, Guangdong, 510006, China; 2 School of Public Administration, Guangzhou University, Guangzhou, Guangdong, 510006, China; 3 Nossal Institute for Global Health, School of Population and Global Health, Carlton, VIC, Australia; 4 School of Management and Director of International Office, Guangzhou University, Guangzhou, Guangdong, 510006, China; 5 School of International Economics and Trade, Shanghai Lixin University of Accounting and Finance, Shanghai, 201209, China; Hainan University, CHINA

## Abstract

Technology-based small and medium enterprises (SMEs) are the driving force behind China’s economic and technological development. However, these enterprises often face challenges in financing their research and development (R&D) activities due to limited financing opportunities. Previous research has primarily focused on the resource attributes of government innovation subsidies, which serve as a crucial funding source for these SMEs. This paper aims to explore the impact of government innovation subsidies on firms from a novel perspective, considering the signaling characteristics of these subsidies. The theoretical foundation of this study lies in the asymmetric information theory and the signaling mechanism through which government subsidies send signals about enterprises. The study uses enterprise data from 2012 to 2019 to investigate the effect of government subsidies on the R&D investment of enterprises listed on the SMEs Board in Chinese stock market. The results reveal a significantly positive effect of government subsidies on the R&D investment of SME Board–listed enterprises and verify the mediating role of financing constraints in this effect. The extent to which government subsidies influence the R&D investment of SME Board–listed enterprises is associated with the enterprises’ ownership characteristics, debt ratios, and times interest earned ratios. This study contributes to the literature on the SMEs Board market and may provide the Chinese government insights into developing industry policies that maximize the effectiveness of government subsidies.

## 1 Introduction

The current wave of globalization is fostering international entrepreneurship, encouraging businesses to seize new environmental opportunities and develop eco-friendly products and services. This, in turn, contributes significantly to global climate protection actions [1–4]. Corporate innovation is widely recognized as the primary force driving both economic development and ecological preservation [5,6]. In response to this, governments worldwide have increased their financial support for corporate innovation.

Enterprise investment in research and development (R&D) refers to an enterprise’s consistent input aimed at upgrading its technology and ensuring its competition advantage in market, which directly influences an enterprise’s technology output in a competitive market and its long-term development planning. To encourage business innovation and growth, governments worldwide have increased their subsidies for R&D financing. With the increasing demand for product funding from technology companies, however, public financing alone is no longer adequate to meet the needs of socioeconomic innovation development, and the “cliff effect” and “welfare trap” of such government subsidies are garnering considerable interest [7,8]. The manner, scope, and timing of public finance intervention in the innovation development of businesses are crucial issues requiring further theoretical and empirical investigation [9–12]. In the realm of entrepreneurship and innovation, market failure in both knowledge and capital markets have emerged due to the disparity between the policy intentions and actual implementation. The failure of the knowledge market can be mitigated by financial resources covering the R&D expenditures of businesses, thereby enhancing their propensity for innovation. Information is the essential component of the capital market, and its failure can be remedied by the efficient application of the information mechanism. The failure of the capital market is the primary contributor to the R&D challenges faced by small and medium enterprises (SMEs).

Theoretically, government funding for enterprises serves two functions: the resource function and the signaling function [13–15]. Government funding for R&D directly augments the resources available to companies, which should incentivize them to boost their investment in innovation. However, according to the “crowding-out effect” and “threshold effect”, if government funding surpasses a certain level, enterprises may become excessively dependent on it. This could displace their own R&D investment and diminish their motivation to secure funds from the market [16,17]. Consequently, the resource function of government innovation funding can be seen as a double-edged sword.

Regarding the signaling function, when a company receives government funding, it signifies that its innovation project has been evaluated and endorsed by the government. This acts as a “recognition label” sending a positive signal to investors. Government recognition not only mitigates the information asymmetry between investors and firms, but it also assists firms in securing more external investment. This alleviates the firm’s financing constraints, thereby increasing R&D investment and gaining additional innovation resources [18,19]. On the other hand, investors may perceive government funding as such firms having a “prototype effect” [20]. Given that technological innovation and start-ups are characterized by long cycles, high investment, and high-risk, government funding for R&D activities may also signal to external investors that the firm has received initial financing and government support, thereby encouraging them to invest in the firm and facilitating external financing, thereby raising the firm’s R & D investment [20].

Financing restrictions can be viewed as a crucial factor in elucidating the distinctions between these two functions. The existence of financing constraints inhibits firms’ R&D investment, and the external financing dilemma they face is more severe than other financing dilemmas [21,22]. From the signaling perspective of government subsidies, the aim of government fiscal innovation funding is to stimulate firms’ R&D investment by alleviating their external financing constraints.

The significance and innovative aspects of this study can be summarized as follows:

Firstly, this paper addresses a crucial research question: Do government subsidies act as a signal? The novelty lies in our investigation of the role that financing constraints play in the relationship between government innovation subsidies and the R&D investment of SMEs. Prior studies on the signaling effect of government subsidies have been limited, and their findings have not always been consistent. To the best of our knowledge, our exploration is not only unique but also holds substantial empirical value by potentially fostering R&D activities within SMEs and enhancing the value of these initiatives. The market’s hesitance to invest in the R&D efforts of innovative firms, due to high costs, long timelines, uncertain R&D outputs, and information asymmetry between firms and their shareholders, underscores the importance of our investigation.

Secondly, our study reconciles the discrepancies in the existing literature by conducting a heterogeneity analysis. This allows us to determine whether the incentivizing impact of government innovation subsidies on corporate innovation varies across different types of firms. It’s important to note that private SMEs often struggle to secure bank loans due to state-owned banks’ preference for state-owned enterprises or larger firms [23]. In a climate of slowing economic growth, structural economic reform, and external financing constraints, government subsidies have emerged as the primary source of external financing for R&D in Chinese SMEs.

Lastly, existing literature tends to conflate the “resource effect” and “signaling effect” of government subsidies [24,25]. Our study not only distinguishes between these two effects but also further refines them based on internal and external financing constraints. This approach aids in shedding more light on the mechanism through which government subsidies signal enterprise innovation. Concurrently, our research reveals that government subsidies can help to alleviate the adverse impact of financing constraints.

By addressing these points, our paper introduces a fresh perspective to the study of government innovation subsidies, examines the role of financing constraints in the relationship between government subsidies and R&D investment of firms, and clarifies the distinct effects of subsidies, thus, presenting a robust and innovative addition to the existing body of literature.

To examine the relationship between government subsidies and the R&D investment of SME Board–listed enterprises, we’ve structured this study into several distinct sections. Section 2 elucidates the pathway and mechanism by which government subsidies, acting as signals, influence firms’ innovation investment. This discussion, grounded in the theories of asymmetric information and signaling, is informed by a comprehensive review of the relevant literature.

In Section 3, we outline the data, variable selection, and empirical model used in our study. We leverage enterprise data from 2012 to 2019 to analyze the impact of government subsidies on the R&D investment of SME Board–listed firms. Our primary methods of analysis include panel fixed-effects (FE), two-way fixed-effects (TWFE), and random-effects (RE) models, which are used to estimate the overall effect of government subsidies on firms’ innovation investment. In addition, we conduct robustness and heterogeneity tests on our empirical models. Furthermore, we employ a mediating effects model to test the transmission mechanism of the signaling effect of government subsidies, with the empirical results presented in Section 4.

The final section is dedicated to a discussion of the results, drawing conclusions, identifying the limitations of this study, suggesting potential avenues for further research, and outlining policy implications. Fundamentally, this study bridges the fields of economics, management theory, and statistical methodology to shed light on the impact mechanism between government subsidies and firms’ innovation investment. It offers a novel research perspective on the signaling properties of government subsidy policies.

## 2 Literature review and analytical framework

### 2.1 Policy effects of government subsidies on firms’ R&D investment

Since Blank and Stigler’s analysis of the relationship between government subsidies and enterprise R&D investment in 1957, the amount of investigations of related topics has increased. Most research has been conducted from one of two major oppositional perspectives: the incentivizing effect [24,26–31] or the crowding-out effect [32–34]. However, no consensus has been reached between the two groups although several scholars have reported an inverted-U relationship between government subsidies and enterprise R&D investment [20,35,36]. The lack of agreement in the literature might be attributable to the existing literature mix-up the resource function and signaling function of government subsidies. Therefore, the question of how government subsidies relate to enterprise R&D investment remains unanswered and with an inconsistent conclusion [37,38].

Previous literature has primarily examined the direct effect of government subsidies on firms’ innovation development from two perspectives: the impact of a single subsidy policy on firms’ innovation development and the impact of multiple subsidy policies in synergy on firms’ innovation development [14,17,20,39–43]. To further investigate the mechanism of government innovation funding and firms’ innovation development, relevant studies have been conducted to investigate the indirect effects of government subsidies on corporate innovation development and to explore the differences in their relationship under the influence of various mediating and moderating factors [44].

Some literature focuses on three levels, namely management level, implementation capability, and external environment, to investigate in greater depth the mechanism by which government subsidies influence innovation investment by firms [45]. For instance, Lei and his team argues that government subsidy actions will adjust firms’ own R&D investment by influencing the innovation willingness and internal incentives of firms’ management boards [46]. In addition, when Quader and Abdullah specifically implemented measures for firms, they discovered that firms’ exploratory learning ability, applied learning ability, and resource absorption ability will cause them to continuously use resources for imitation and application, which will impact firm innovation [47]. Finally, Chen and his team examines the external environment and concludes that society’s intermediaries and the manner in which government power is exercised can play a significant role in mediating the relationship between government grants and firm innovation [48].

In addition to the mediating role, the indirect effect of government subsidies on firm innovation is moderated by several factors. These moderating variables can be grouped broadly into three main categories: fundamental characteristics of the firm, the current state of firm management, and external business environmental factors [49]. For instance, Beaudry et al. discovered that different firm types, firm size, and firm nature (whether it is a state-owned enterprise) moderate the relationship between government subsidies and firm innovation [50]. In addition, Baernanke and Kuttner find that the government’s enforcement of IPR protection regulations, executives’ awareness of innovation program, and the intensity of R&D investment all play important moderating roles [51]. Zhang and his team concludes that the external environment, such as the level of economic development, the degree of market distortion, the income tax system, and the credit system in various regions, also play a significant moderating role in the relationship between government subsidies and firms innovation development [52].

### 2.2 Theoretical foundations of the signaling effect of government subsidies

The asymmetric information between entrepreneurs and external investors is, in fact, the greatest obstacle for small and medium-sized R&D companies. The knowledge assets of small and medium-sized R&D firms are so risky, less traditional collateral, and proprietary that an unusual capital market failure problem arises. The R&D investment problem of such companies necessitates a greater focus on the signaling function property of government subsidies.

The signaling function theory emphasizes that government R&D grants can send positive signals about a company to market investors, thereby easing that company’s financing constraints [48]. Under the signaling mechanism of government grants, market investors’ recognition and evaluation of the enterprise will improve based on the additional certification of such grants, the enterprise’s financing constraints will be relieved, external financing channels will expand, and the enterprise’s R&D financing will increase [53]. Thus, it is evident that government innovation grants influence firms’ R&D investments via the mechanism of financing constraints.

In fact, financing constraint has been widely studied as an important factor to measure the cost of internal and external financing of firms. Greenwald, Stigliz, and Weiss defined it as the difference between the existence of internal and external financing costs of firms due to the imperfection of capital markets (asymmetric information, agency costs, etc.) [54]. Brown, Fazzari, and Petersen provide empirical evidence that the issue of financing restrictions on corporate innovation exists in all the world’s major economies. The financing constraint plays a significant role in mediating their relationship [16].

**Fig 1:**
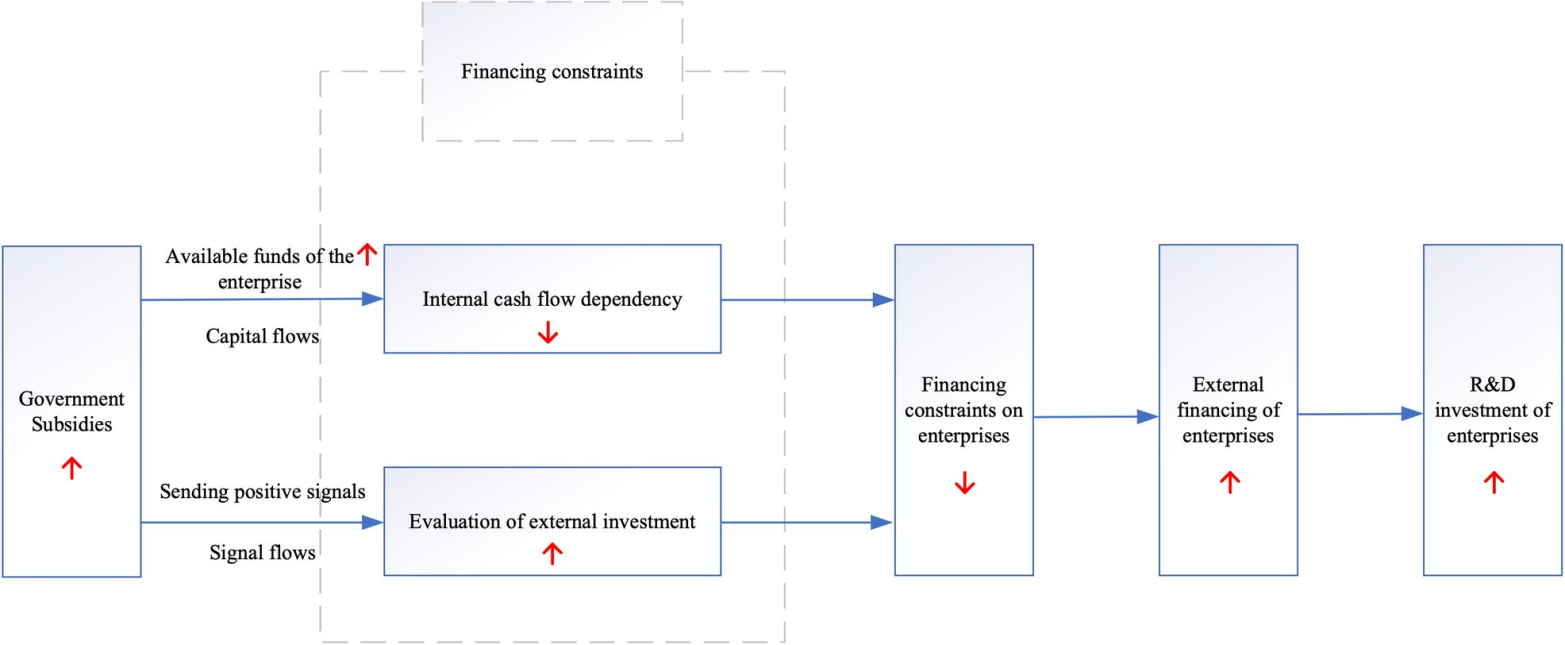
Analytical framework of influence mechanism of financing constraints.

Previous studies have demonstrated that government subsidies alleviate financing constraints in enterprise R&D, thus encouraging enterprise R&D investment. This relationship can be summarized and illustrated in Fig 1.

Existing research has indeed investigated the influence of government subsidies on the R&D investment of enterprises, with some studies considering the impact of financing constraints on this relationship [15,55–57]. However, there is a distinct lack of analyses that view financing constraints as a mediating factor in this dynamic. Moreover, the degree to which these constraints affect the relationship, particularly in the context of enterprises listed on the SME Board, remains largely unexplored. Our study seeks to address these gaps by focusing specifically on SME Board–listed enterprises. We aim to elucidate the extent to which government subsidies stimulate enterprise R&D investment and how this stimulation is mediated by the alleviation of financing constraints. To further refine our analysis, we consider ownership characteristics, debt ratios, and times interest earned ratios as crucial determinants of the degree of R&D financing constraints faced by these enterprises. Such factors, in turn, shed light on the effect of financing constraints on the interplay between government subsidies and enterprise R&D investment.

R&D investment is intrinsically tied to how enterprises allocate their resources. Within the framework of our study, we explore the mechanisms through which internal and external constraints, primarily financing constraints, influence this relationship. This approach provides a fresh perspective and deeper understanding of the dynamics at play.

## 3 Method

### 3.1 Data and variables

The preponderance of the data utilized in this study is derived from the RESSET and China Stock Market (CSMAR) databases, with government subsidy information procured from the “government subsidies included in current profit and loss” indicator in the annual reports of the respective companies.

Detail description of these two datasets can be found elsewhere [58–61]. From these two datasets, we know that Chinese innovation subsidies encompass a wide range of categories, covering an extensive scope. This includes program-based innovation subsidies such as the National High Technology R&D Program (863 Program) and the Torch Program, as well as recognition-based innovation subsidies like high-tech enterprise recognition and patent pilot demonstration enterprise recognition. Additionally, funding for technology projects, such as the Special Program for Independent Innovation of Enterprises and the Technology Innovation Fund for Small and Medium-sized Enterprises, provides subsidies for eligible enterprises and their R&D projects under different programs, recognition methods, and funds. For instance, by the end of 2018, the Technology Innovation Fund for Small and Medium-sized Enterprises in Science and Technology had allocated a cumulative central financial budget of 26.826 billion yuan, supporting 46,282 projects. Cumulative data from 13,901 projects supervised by 37 local science and technology authorities and provincial-level supervisory units showed that 86.97% of the technology during the project execution period came from the enterprises undertaking the projects, and the enterprise R&D expenditure accounted for 11.02% of the total annual revenue. Furthermore, 7,319 projects were granted patents, with 3,718 projects receiving invention patents. The total annual revenue, annual export value, and annual tax payment of the enterprises increased by 52.56%, 67.94%, and 61.92%, respectively, before and after the project establishment.

In this study, SMEs listed in the databases are chosen as the research sample for two reasons: first, extant literature exhibits a dearth of studies concerning related topics with SMEs as the sample; and second, SMEs constitute the driving force behind R&D innovation in the prevailing economic climate, and the sustenance of R&D investment in SMEs is an indispensable factor in preserving market vitality and fostering sustainable economic development within a nation.

This study selects valid observations in accordance with the following criteria: first, financial companies within the sample are excluded, which include banks, credit unions, brokerages, investment firms and so on; second, companies experiencing two consecutive years of losses (ST) and those with three and more consecutive years of losses (*ST) are omitted; and third, companies with missing years or critical variables are excluded. The final panel data encompasses 2,960 observations from 370 SMEs listed on the SME Board–listed from 2012 to 2019 (all data are recorded for the actual financial year of 2011 to 2018). The descriptive statistics of the primary variables are presented in Table 1.

**Table 1:** Descriptive statistics, 2012–2019.

Variables	Label	OBS	Mean	S.D.	Min	Max
*LnRD*	Firm’s R&D investment	2960	17.8931	1.2068	12.3559	22.2235
*LnGS*	Government innovation subsidies	2960	15.9998	1.2578	9.2103	20.7593
*S*	Growth rate of operating income	2960	0.2516	1.9469	-0.8007	84.992
*ROE*	Return on equity	2960	0.0507	0.0631	-0.6476	0.5981
*Size*	The enterprise scale	2960	21.8631	0.8776	19.1987	25.5225
*OC*	Degree of ownership concentration	2960	0.3288	0.1436	0.0415	0.8649
*Q*	Tobin’s Q value	2960	2.2331	1.5002	0.4673	31.4002

Sources: RESSET database and China Stock Market (CSMAR) database, 2012–2019.

### 3.2 Ethics statement

We obtained permission to use the publicly accessible business and stock market datasets from the Ruisi RESSET Dataset and the CSMAR Database. Access to these two datasets is granted upon registration and purchase. The datasets do not involve any human subjects, and all non-publicly available human identifiable information related to the companies was removed upon receipt of the data. Consequently, this study no longer necessitates ethics approval or informed consent.

### 3.3 Empirical model

This paper explores whether there is a positive correlation between Chinese government financial innovation subsidies and firms’ R&D investment in China. From the perspective of the resource function attribute, due to the existence of the crowding-out effect and rent-seeking behavior by firms, government subsidies do not necessarily lead to an increase in firms’ R&D investment. However, from the perspective of the signaling functional attribute, government subsidies can induce social investment to follow suit. Through financial replenishment, shifts in innovation propensity, and complementary supervision, these three interactions can collectively contribute to an increase in the overall R&D investment of enterprises [62–70].

Based on the existing literature, the FE and TWFE model is commonly used in the empirical analysis [14,15,47,71]. Therefore, the following baseline model is proposed:

$$ln{RD}_{it}=\alpha_{i}+\beta_{1}{GS}_{it-1}+\beta X_{it}+u_{i}+v_{t}+\varepsilon_{it}$$ (1)

where ln*RD*_*it*_ represents the logarithm of the total amount R&D investment (in RMB) of firm *i* in year *t*. *GS*_*it-1*_ refers to the amount of government innovation subsidies (log-transformed) that the *i*-th firm received in year *t*. These subsidies information are collected from the period after an enterprise’s income statement contains government subsidies, has also been indicated in the enterprise’s annual financial report. These data are used to determine the lagging effect of government subsidy policy (*t-1*). In a robustness test, the ratio of government subsidies to sales revenue within a year is employed as a proxy variable for such government funding. *X*_*it*_ is a series of control variables that include net return on equity (*ROE*), enterprise size (*Size*), ownership concentration (*OC*), investment opportunities for enterprises (*Q*), and enterprise growth (*S*).

Eq (1) can be used to determine the direct effect of government innovation subsidies for innovation activities on enterprise R&D input. To further explore the mediating role of financing constraints in the relationship between government innovation subsidies and the R&D investment of SME Board–listed enterprises, this study employs Guo’s approach and categorizes enterprises into two main groups: state-owned and private-owned [72]. In line with the studies of Lu et al. and Lian et al., enterprises are ranked by their debt ratios and times interest earned ratios; enterprises in the top and bottom 30% are identified as enterprises with low-financing and high-financing constraints, respectively [73,74].

To investigate the effect of financing constraints on the relationship between government innovation subsidies (*GS*) and enterprise R&D investment (*lnRD*), this study follows the methodologies of previous research and includes a mediator—financing constraints (*CF*) and an interaction term between government innovation subsidies and financing constraints (GS^*^CF) in the baseline model to determine the mediating effect of internal and external financing constraints. The modified equation is as follows:

$${lnRD}_{it}=\alpha_{i}+\beta_{1}{GS}_{it-1}+\beta_{2}{CF}_{it}+\beta_{3}{CF}_{it}*{GS}_{it-1}+\beta X_{it}+u_{i}+v_{t}+\varepsilon_{it}$$ (2)

where *β*_*3*_ is the coefficient of the interaction term; a significant interaction term is considered to verify the mediating effect of financing constraints. Specifically, the internal financing constraints are determined by net cash flow created by an enterprise’s business activities, while external financing constraints are measured using financial indicators in accordance with Lin et al. and Kuang et al., respectively [21,75,76].

## 4 Results

### 4.1 Descriptive statistics

**Fig 2:**
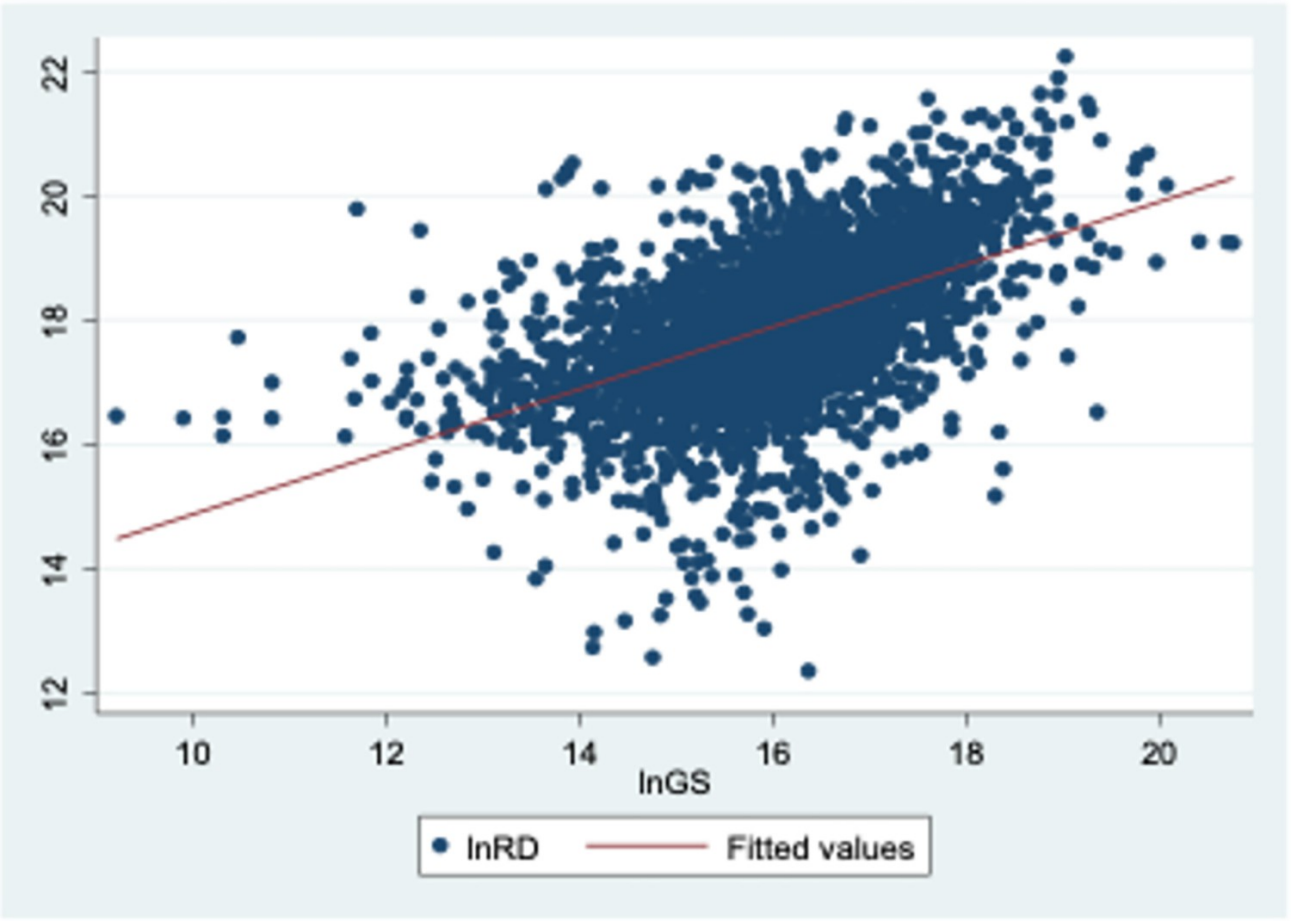
The correlation between enterprise R&D investment and government innovation subsidy. Sources: RESSET database and China Stock Market (CSMAR) database, 2012–2019.

Table 1 presents the descriptive statistics of the main variables while Fig 2 below illustrates the relationships between government innovation subsidies and R&D investment in all sample enterprises from 2012 to 2019. The horizontal axis is the core explanatory variable (log-transformed total amount of government innovation subsidies), while the vertical axis is the explanatory variable (log-transformed of firms’ R&D investment). The descriptive statistics in Table 1 provides a clear picture of the innovation inputs of firms, represented by the logarithm of firms’ innovation inputs (*LnRD)*. The mean *LnRD* is approximately 17.89, indicating a general trend among firms to invest in R&D activities, predominantly of a lower technological level. However, the S.D. value of 1.2068 suggests substantial variation in the level of innovation inputs across different firms. This is further reinforced by the range of the *LnRD* values, with the minimum and maximum values at 12.3559 and 22.2235 respectively. These findings align with the diverse innovation landscape of Chinese firms, which encompasses both low-end innovation and high-end R&D.

Regarding government innovation subsidies, represented by the logarithm of government subsidies (*LnGS*), the mean subsidy level is 15.9998. This figure suggests that government funding accounts for approximately 89% of the firms’ R&D investment, indicating a high level of support from the Chinese government. However, the S.D. value of 1.2578 and the range of *LnGS* values, from a minimum of 9.2103 to a maximum of 20.7593, reveal considerable disparities in the subsidies received by different enterprises. This variation aligns with the actual conditions of the Chinese R&D landscape, thereby validating the reliability of our dataset.

The scatter plot analysis further suggests a significant positive correlation between government innovation subsidies and enterprises’ R&D investment, the higher the government innovation subsidies, the greater the R&D investment. Given these findings, we believe it is worthwhile to further explore the role of government innovation subsidies in this context. Moreover, for the moderating variables, such as the operating income growth rate, return on net assets, firm size, equity concentration, and Tobin’s Q are all related to firm R&D investment. Therefore, they are included in the group of control variables in the subsequent empirical model. The following section describes the regression analysis used to determine the significance of the relationship between enterprise R&D investment and government innovation subsidies.

### 4.2 Regression analysis

In this study, we employ fixed-effects (FE), two-way fixed-effects (TWFE), and random-effects (RE) models to generate the empirical results presented in Table 2. Models 1a and 2a function as baseline regression models, with Model 1a excluding and Model 2a including control variables. Models 3a and 4a are TWFE regression models; they differ from Models 1a and 2a by incorporating both individual and time fixed-effects. Models 5a and 6a are employed for robustness testing: in Model 5a, we used R&D intensity (RDI) as the dependent variable instead of R&D investment (*LnRD*), and Model 6a utilizes a RE model instead of TWFE model used in Model 4a.

**Table 2:** Regression analysis, FE, TWFE and RE models, full sample.

Variables	(Model 1a)	(Model 2a)	(Model 3a)	(Model 4a)	(Model 5a)	(Model 6a)
	LnRD	LnRD	LnRD	LnRD	RDI	LnRD
*LnGS*	0.325***	0.0889***	0.131***	0.0724***	0.913***	0.101***
	(24.82)	(7.84)	(10.57)	(6.40)	(4.04)	(9.05)
*S*		0.0107*		0.0146**	0.151	0.00996*
		(2.27)		(3.12)	(1.62)	(2.11)
*ROE*		0.317		0.645***	13.23***	0.458*
		(1.74)		(3.49)	(3.58)	(2.55)
*Size*		0.798***		0.674***	-5.232***	0.801***
		(38.86)		(23.93)	(-9.29)	(41.31)
*OC*		-1.046***		-0.646***	-12.15***	-0.874***
		(-6.19)		(-3.63)	(-3.42)	(-6.00)
*Q*		0.0238**		0.0229*	0.862***	0.0280***
		(3.05)		(2.47)	(4.65)	(3.63)
Constant term	12.70***	-0.715	15.22***	1.914**	123.3***	-1.032**
	(60.54)	(-1.68)	(79.02)	(3.25)	(10.47)	(-2.62)
*N*	2960	2960	2960	2960	2960	2960
Model specification	FE	FE	TWFE	TWFE	TWFE	RE
Individual FE	*Y*	*Y*	*Y*	*Y*	*Y*	*N*
Time FE	*N*	*N*	*Y*	*Y*	*Y*	*N*
R^2^	0.1923	0.5443	0.4353	0.5602	0.0763	
F/WALD	616.26	514.38	248.80	252.48	16.38	3328.34

Sources: RESSET database and China Stock Market (CSMAR) database, 2012–2019.

Notes: 1) FE: Fixed–effect model, RE: Random–effected model; TWFE: Two–ways fixed–effect. 2) the value of t–test in parentheses, sig: ^*^ p<0.1, ^**^ p<0.05, ^***^ p<0.01.

According to the regression results, the *p* values corresponding to the *F* statistics of all models are less than 0.001, indicating significance at the 99% confidence level. Therefore, the model configurations are valid. Table 2 reveals that the coefficient of *lnRD* is 0.325 in Model 1a (*p* = 0.000), suggesting that with every 1% increase in government innovation subsidies, enterprises increase R&D input by 0.325%. In Model 3a, where both time and individual FE are employed (also known as the TWFE model), the coefficient of *lnGS* is decreased to 0.131 (*p* = 0.000). The results reveal a positive effect of government innovation subsidies on the R&D investment of SMEs Board–listed enterprises.

For Model 2a, which contains control variables, the coefficients for operating income growth (*S*), enterprise size (*Size*), and the Tobin’s Q ratio (*Q*) are all significantly positive. The results indicate that quick growth, large enterprise size, and presence of abundant investment opportunities are all drivers of SME Board–listed enterprises engaging in R&D activities. The coefficient for net *ROE* is insignificantly positive, suggesting that the effect of *ROE* on R&D investment is nonsignificant; this may be due to heterogeneity in the industry or enterprise leading to considerably different findings regarding the effects of enterprise earnings on R&D activities. The coefficient for *OC* is significantly negative at the 99% confidence level, which is opposite to the prediction of this study; this negative association is probably the result of private SMEs being less active in investing. In Model 4a the TWFE model shows that the significance levels of the control variables increase; this result verifies the presence of enterprise heterogeneity. The effect of earnings on R&D investment, despite varying among the SME Board–listed enterprises, is positive.

The coefficients for the variables change as how the dependent variable is measured and how the model is set change. However, the relationship between government innovation subsidies (*lnGS*) and enterprise R&D investment (*lnRD*) remains significant across the changes. The result of Model 5a further verifies the significantly positive effect of government innovation subsidies on SMEs’ R&D investment and validates the robustness of the empirical results. In a word, the empirical investigation reveals a significantly positive effect of government subsidies on the R&D investment of SME Board–listed enterprises.

### 4.3 Heterogeneity analysis

#### 4.3.1 Rationale for heterogeneous grouping

In order to ascertain the role and influence mechanism of the moderating variables between the dependent and independent variables, this section employs three factors—corporate equity nature, gearing level, and interest cover size—as grouping indicators, subsequently conducting a heterogeneity analysis grounded in the baseline model. Notably, Xu and other coauthors contended that the impact of financial market development on fostering corporate R&D investment is subject to local government policy interventions, which in turn exacerbate corporate financing constraints. This is attributed to China’s economic system markedly diverging from other open economies. Their research categorizes state-owned SMEs as the “weak financing constraint group” and private-owned SMEs as the “strong financing constraint group” [77,78]. Zhang et al. characterize the top 30% of firms’ gearing as the “weak financing constraint group” and the bottom 30% as the “strong financing constraint group” [52,79]. In line with previous studies, the lower the interest coverage multiple, the higher the probability of debt default [14,80]. Consequently, the largest 30% of interest coverage multiples are defined as the “weak financing constraint group” and the smallest 30% as the “strong financing constraint group”.

The primary objective of this paper is to examine the mediating effect of financing constraints in the process of government innovation subsidies promoting R&D investment in listed SMEs. The aim is to analyze the influence mechanism between government innovation subsidies and R&D investment in listed SMEs from a financing constraint perspective. Table 3 presents the results of the heterogeneity analysis based on Model 4a.

**Table 3:** Heterogeneity analysis, weak and strong financing constraints subsamples.

	Weak financing constraints			Strong financing constraints		
	(Model 1b)	(Model 2b)	(Model 3b)	(Model 4b)	(Model 5b)	(Model 6b)
	Small- and medium-sized state-owned enterprises	High debt-asset ratio	High interest coverage multiple	Small- and medium-sized private-owned enterprises	Low debt-asset ratio	Low interest coverage multiple
	*LnRD*	*LnRD*	*LnRD*	*LnRD*	*LnRD*	*LnRD*
*LnGS*	0.121***	0.0958***	0.0726***	0.0944***	0.0362	0.0349
	(3.57)	(3.59)	(3.33)	(7.64)	(1.89)	(1.69)
*S*	0.035	0.0272*	-0.00479	0.0109*	0.0127	0.0433
	-0.92	-2.23	(-0.72)	(2.41)	(1.81)	(1.2)
*ROE*	-0.034	0.975*	0.347	0.159	0.407	0.386
	(-0.05)	(1.99)	(1.01)	(0.78)	(1.37)	(1.12)
*Size*	0.935***	0.829***	0.822***	0.768***	0.772***	0.770***
	(14.99)	(15.22)	(22.09)	(35.49)	(19.41)	(18.25)
*OC*	-0.63	-1.470***	-0.922**	-1.098***	-1.205***	-1.253***
	(-1.08)	(-3.54)	(-2.61)	(-6.03)	(-4.06)	(-4.17)
*Q*	0.0710**	0.0318	-0.00043	0.0244**	0.0165	0.0354**
	(2.68)	(1.08)	(-0.03)	(2.97)	(1.76)	(3.03)
Constant term	-4.499***	-1.505	-0.79	-0.123	0.753	0.798
	(-3.46)	(-1.31)	(-1.00)	(-0.27)	(0.87)	(0.92)
*N*	498	888	888	2242	888	888
Model specification	*TWFE*	*TWFE*	*TWFE*	*TWFE*	*TWFE*	*TWFE*
R^2^	0.4889	0.4495	0.5536	0.5755	0.5111	0.5165
*F-test*	105.22	91.87	128.55	439.72	116.72	105.22
Individual effect	significant	significant	significant	significant	significant	significant

Sources: RESSET database and China Stock Market (CSMAR) database, 2012–2019.

Notes: 1) TWFE: Two–ways fixed–effect. 2) the value of t–test in parentheses, sig: ^*^ p<0.1, ^**^ p<0.05, ^***^ p<0.01.

#### 4.3.2 Heterogeneity analysis in financing constraints

The following subsections present the discussions and conclusions of the analyses conducted when the sample was grouped based on financing constraints:

### (1) Significant mediating effect of financing constraints

According to the subsample regression results, the effect of government innovation subsidies on R&D investment is more significant in the weak financing constraint group than it is in the strong financing constraint group. This result suggests that financing constraints have a significant influence on the extent to which government innovation subsidies encourage R&D investment in SME Board–listed enterprises.

### (2) SME Board–listed enterprises with different ownership characteristics

The results obtained after ownership-characteristic grouping reveal that the *lnGS* coefficient is greater for state-owned SME Board–listed enterprises than it is for private-owned (Model 1b and Model 4b). This is possibly because external R&D investors tend to prefer investing in state-owned, subsidized enterprises over investing in private enterprises. These investors also exhibit a higher preference for larger enterprises because such enterprises are exposed to relatively low risks. Accordingly, financing constraints on R&D investment are higher in private SME Board–listed enterprises than they are in state-owned enterprises.

### (3) SME Board–listed enterprises with different debt ratios

The results obtained after grouping by debt ratio show that the coefficient for *lnGS* is significantly positive in Model 2b and insignificantly positive in Model 5b. This indicates that enterprises with high debt ratios face lower financing constraints; thus, the effect of government innovation subsidies on R&D investment is greater for these enterprises.

### (4) SME Board–listed enterprises with different times interest earned ratios

A comparison of SMEs with high and low times interest earned ratios reveals a significant influence of government innovation subsidies on the innovation investment of SMEs with high times interest earned ratios (Model 3b and Model 6b). This effect is insignificant among SMEs with low times interest earned ratios. Therefore, times interest earned ratios can be used to determine the level of financing constraints on SMEs and significantly affect the extent to which government innovation subsidies increase the R&D investment of SME Board–listed enterprises.

In conclusion, this result indicates that the positive effect of government innovation subsidies on the R&D investment of SME Board–listed enterprises is mediated by financing constraints and affected by the ownership characteristics, debt ratios, and times interest earned ratios of enterprises.

#### 4.3.3. Mechanism analysis between government innovation subsidies and enterprise R&D investment

Studies have mostly reported a significantly positive effect of the amount of funds enterprises allocate to R&D activities. Lu and his team suggests that the cash holdings of an enterprise can offset the sensitivity of R&D investment to cash flow [75]. Therefore, in the present study, the net cash flow generated through the business activities of SMEs is employed as an indicator of internal financing constraints (*CF1*).

Table 4 presents the empirical results related to internal financing constraints. Models 1c and 2c are baseline regression models without and with control variables, respectively; for both models, individual and time effects are fixed. Model 3c is the baseline regression model for Model 3c. Model 4c is established by fixing the time and individual effects in Model 3c. Model 5c is based on Model 2c, with *lnRD* replaced by *RDI* for measuring enterprise R&D investment and is used for robustness testing. Model 6c is another internal constraint model based on Model 2c, with changes made to the RE model.

**Table 4:** Endogenous mediating mechanism analysis, full sample.

	(Model 1c)	(Model 2c)	(Model 3c)	(Model 4c)	(Model 5c)	(Model 6c)
	LnRD	LnRD	LnRD	LnRD	RDI	LnRD
*LnGS*	0.131***	0.0724***	0.0788***	0.0589***	0.0589***	0.0920***
	(10.57)	(6.40)	(6.73)	(5.03)	(5.03)	(7.97)
*CF1*			-0.0517**	-0.0729***	-0.0730***	-0.0445*
			(-2.67)	(-3.77)	(-3.77)	(-2.30)
*LnGS* ^*^ *CF1*			0.00319**	0.00432***	0.00432***	0.00278*
			(2.94)	(3.99)	(3.99)	(2.58)
*S*		0.0146**	0.0150**	0.0212***	0.0212***	0.0135**
		(3.12)	(2.97)	(4.22)	(4.22)	(-2.67)
*ROE*		0.645***	0.265	0.645***	0.645***	0.401*
		(3.49)	(1.43)	(3.43)	(3.43)	(2.19)
*Size*		0.674***	0.786***	0.655***	-0.345***	0.789***
		(23.93)	(36.9)	(22.83)	(-12.04)	(39.01)
*OC*		-0.646***	-1.085***	-0.669***	-0.668***	-0.899***
		(-3.63)	(-6.42)	(-3.78)	(-3.77)	(-6.18)
*Q*		0.0229*	0.0225**	0.0185*	0.0185*	0.0266***
		(2.47)	(2.88)	(1.99)	(1.99)	(3.45)
Constant term	15.22***	1.914**	-0.265	2.545***	9.455***	-0.619
	(79.02)	(3.25)	(-0.59)	(4.18)	(15.53)	(-1.48)
*N*	2960	2960	2960	2960	2960	2960
Model specification	TWFE	TWFE	FE	TWFE	TWFE	RE
Individual FE	Y	Y	Y	Y	Y	N
Time FE	Y	Y	N	Y	Y	N
*R* ^*2*^	0.4353	0.5602	0.5469	0.5635	0.0951	
*F/WALD*	248.80	252.48	389.52	221.63	18.04	3354.79

Sources: RESSET database and China Stock Market (CSMAR) database, 2012–2019.

*Notes*: *1) FE*: *Fixed–effect model*, *RE*: *Random–effected model; TWFE*: *Two–ways fixed–effect*. *2) the value of t–test in parentheses*, *sig*: ^*^ *p<0*.*1*, ^**^ *p<0*.*05*, ^***^ *p<0*.*01*.

The *p* values corresponding to the *F* statistics of all models are <0.001, indicating that the models are significant at the 99% confidence level. The model settings are thus reasonable and meaningful.

An analysis of the mediating effect of *CF1* reveals that the mediators (*CF1*) and interaction terms (lnGS^*^CF1) are significant in several models. This verifies the mediating role of internal financing constraints. Additionally, Models 4 and 2, in which time and individual effects are fixed, have positive coefficients for the interaction term; this suggests that enterprise R&D investment increases as the enterprise’s internal funds increase. Therefore, internal funds alleviate the effect of internal financing constraints on the relationship between government innovation subsidies and enterprise R&D investment.

Models 5c and 6c are both used to test the robustness of the models based on Model 2c, with the dependent variable replaced and model settings changed, respectively. The results of Model 2c, both before and after it is changed to Models 5c and 6c, are significant, supporting the reliability and reasonableness of the empirical results.

#### 4.3.4 Analysis of external financing constraints

This study conducts a multiple discriminant analysis of the debt ratios (*D*_*t*_), current ratios (*Currt*), and working capital ratios (*W*_*t*_) of SME Board–listed enterprises from 2012 to 2019 based on previous studies [71,73,76,81]. Subsequently, the positive and negative signs of the coefficients are reversed. A moderating indicator (*CF2*_*it*_) is developed to measure the external financing constraints faced by SME Board–listed enterprises. The equation is as follows:

$${CF2}_{it}=2.387-6.232{Dt}_{it}-0.027{Currt}_{it}-0.202{Wt}_{it}$$ (3)

The external financing constraints indicator (*CF2*_*it*_) is subsequently substituted into the external constraint model to obtain the results presented in Table 5. Models 1d and Model 2d are baseline regression models without and with control variables, respectively; in both models, the individual and time effects are fixed. Model 3d is the baseline regression model of Model 2d. Model 4d is based on Model 3d. However, in Model 4d, the time and individual effects are fixed. Model 5d is based on Model 2d. However, in Model 5, the enterprise R&D investment indicator *lnRD* is replaced with *RDI* for robustness testing. Model 5 is another internal constraint model based on Model 2, with changes made to the model settings to test Model 2’s robustness.

**Table 5:** Exogenous mediating mechanism analysis, full sample.

	(Model 1d)	(Model 2d)	(Model 3d)	(Model 4d)	(Model 5d)	(Model 6d)
	LnRD	LnRD	LnRD	LnRD	RDI	LnRD
*LnGS*	0.131***	0.0724***	0.0876***	0.0712***	0.0712***	0.0995***
	(10.57)	(6.4)	(7.71)	(6.29)	(6.29)	(8.9)
*CF2*			0.380**	0.336*	0.336*	0.314*
			(2.66)	(2.39)	(2.38)	(2.23)
*LnGS* ^*^ *CF2*			-0.0230*	-0.0200*	-0.0200*	-0.0181*
			(-2.54)	(-2.24)	(-2.24)	(-2.03)
*S*		0.0146**	0.0113*	0.0151**	0.0151**	0.0106*
		(3.12)	(2.39)	(3.23)	(3.23)	(2.24)
*ROE*		0.645***	0.299	0.613**	0.614**	0.405*
		(3.49)	(1.59)	(3.21)	(3.22)	(2.18)
*Size*		0.674***	0.801***	0.680***	-0.320***	0.809***
		(23.93)	(36.37)	(22.83)	(-10.74)	(38.79)
*OC*		-0.646***	-1.033***	-0.635***	-0.634***	-0.850***
		(-3.63)	(-6.06)	(-3.56)	(-3.56)	(-5.80)
*Q*		0.0229*	0.0239**	0.0231*	0.0231*	0.0282***
		(2.47)	(3.05)	(2.48)	(2.48)	(3.66)
Constant term	15.22***	1.914**	-0.751	1.792**	8.700***	-1.196**
	(79.02)	(3.25)	(-1.64)	(2.87)	(13.94)	(-2.81)
*N*	2960	2960	2960	2960	2960	2960
Model specification	FE	TWFE	FE	TWFE	TWFE	RE
Individual FE	Y	Y	Y	Y	Y	N
Time FE	Y	Y	N	Y	Y	N
*R* ^*2*^	0.4353	0.5602	0.5456	0.5613	0.0905	
*F/WALD*	248.80	252.48	387.58	219.64	17.08	3341.96

Sources: RESSET database and China Stock Market (CSMAR) database, 2012–2019.

*Notes*: *1) FE*: *Fixed–effect model*, *RE*: *Random–effected model; TWFE*: *Two–ways fixed–effect*. *2) the value of t–test in parentheses*, *sig*: ^*^ *p<0*.*1*, ^**^ *p<0*.*05*, ^***^ *p<0*.*01*.

The *p* values corresponding to the *F* statistics of all models are <0.001, indicating these models are significant at the 99% confidence level and the model settings are reasonable and meaningful.

The mediator (*CF2*) and interaction term (lnGS^*^CF2) remain significant across several models, supporting that external financing constraints have a mediating effect. In addition, the coefficients of the interaction terms in Models 4d and Model 2d, in which time and individual effects are fixed, are significantly negative. This suggests that the extent to which government innovation subsidies encourage SME R&D investment is influenced by the level of external financing constraints and is reduced by said constraints.

Models 5d and Model 6d are models based on Model 2d, with the dependent variable replaced and model settings changed, respectively. No substantial change is noted in the results following these changes. Therefore, the results regarding the external financing constraints are robust.

## 5 Conclusion and policy recommendations

Grounded in asymmetric information theory and the signaling mechanism and drawing on the FESSET and CSMAR databases from 2012 to 2019, this paper investigates the impact of government innovation subsidies on the R&D investment of SMEs Board–listed enterprises. The empirical results reveal three key findings, derived from a fixed panel effects model combined with a mediation effect analysis. First, financial R&D grants in China demonstrate a signaling function effect. The empirical evidence indicates that a 1% increase in government innovation subsidies (*GS*) leads to a 0.325% rise in firms’ R&D investment (*RD*), signifying a significant positive correlation primarily driven by the signaling mechanism. Second, the crucial mediating variable enabling this signaling function is the financing constraint faced by firms. The influence of government innovation subsidies on firms’ R&D investment manifests in two ways: on one hand, it appears in the resource effect, which reduces the sensitivity to cash flow within the firm. The empirical results show that the elasticity of the interaction coefficient between *GS* and internal financing constraints is 0.00319, surpassing the 1% significance threshold. Conversely, it is evident in the demonstration effect, which alleviates the external financing constraint of enterprise R&D. The empirical findings demonstrate that the elasticity of the interaction coefficient between government innovation subsidies and external financing constraints is -0.0230, exceeding the 10% significance threshold. Finally, substantial firm heterogeneity exists regarding the performance of the signaling function of financial R&D grants in China. The empirical results from the sub-sample indicate that although the policy effect is significant for both state-owned and non-state-owned enterprises, disparities persist (with elasticity coefficients of 0.121 and 0.0944, respectively). The policy effect is significant for the group with a high level of corporate assets and liabilities, yielding an elasticity coefficient of 0.0958 and surpassing the 1% significance threshold; however, the policy effect is not significant for the group with low assets and liabilities. The policy effect is significant for the group with large interest coverage multiples, exhibiting an elasticity coefficient of 0.0726, which exceeds the 1% significance threshold, while the policy effect for the group with small interest coverage multiples is insignificant. The following policy suggestions are proposed based on the empirical results.

First, the level of government innovation subsidies should be modified based on the current state of market development to encourage SME R&D investment in general. Government subsidies have a generally positive impact on the R&D expenditures of SME Board–listed companies and alleviate the financing constraints these companies face [20,35,36]. Consequently, it is necessary to identify the market’s needs and adjust based on the magnitude of government innovation subsidies to maximize subsidies and facilitate R&D activities in SMEs.

Second, for the government, when the financing constraint of enterprises is strong to a certain degree, government grants have limited incentive utility for enterprises’ R&D investment, and the signal function of government grant behavior should be maximized to direct social investors to follow up [14,17,20,39–43]. The signal function is dependent on the credibility of government subsidy policy, which necessitates the improvement of the ex-ante review, ex-post supervision, and information disclosure mechanism of government subsidy behavior [14,20,40]. On the one hand, the reliability, quality, and promising characteristics of the funded projects can be communicated to the public through an open and transparent monitoring mechanism. On the other hand, the post-grant review and monitoring mechanism should be strengthened to prevent the “crowding-out effect” of enterprises’ R&D investment and “strategic” R&D direction, thereby boosting public confidence in enterprises’ original innovation [17,40,41].

Lastly, enterprises should maximize the positive signal function property made available by government grants to bolster their own R&D financing channels and amass more resources to promote R&D investment [46]. On the one hand, improve the internal supervision mechanism of enterprises, strengthen the effective monitoring of the process of using government grants, improve the utilization rate of funds in innovation investment, increase the level of enterprise innovation, and bolster the enterprises’ core competitiveness [47]. On the other hand, all public financial indicators, including operating ability, profitability, and income and expenditures, are crucial for attracting foreign investment [48]. Therefore, enterprises should focus on improving their achievements in these areas, seizing, and attracting valuable R&D financing opportunities at the appropriate time, and actively introducing social investment institutions so that both parties can discuss, construct, and share to boost the overall innovation level of enterprises.

Nonetheless, the present study possesses limitations that warrant attention in subsequent research. First, the definitions of empirical variables necessitate refinement. This paper employs total government funding received by firms during the current period as a proxy for government innovation funding, without distinguishing between asset-related, revenue-related, and other categories of government funding. Consequently, future research should investigate government funding on a project-specific basis to attain a more comprehensive comprehension of its signaling function. Second, due to data constraints, this study does not differentiate between substantive and strategic innovation, or between invention patents, utility model patents, design patents, and so on, which is particularly notable in the context of Chinese firms. The empirical model assumes a linear relationship between government funding and corporate R&D investment, utilizing a panel FE and TWFE model as well as interaction terms to validate the transmission mechanism. In future research, this assumption could be relaxed to ascertain whether a nonlinear relationship, such as an inverted-U-shaped relationship, exists between the two variables. The subsequent research goal would be to identify a more effective method for examining the mediating effect that elucidates the role of financing constraints.

## Supporting information

S1 Data (ZIP)

## Abbreviations

CSMAR: China Stock Market
FE: Fixed-effect
OC: Ownership concentration
RE: Random-effect
R&D: Research and development
RDI: Research and development intensity
ROE: Return on equity
SMEs: Small and Medium Enterprises
TWFE: Two-ways Fixed-effect
