# Expression of Concern: How innovation funding leads enterprises to engage in research and development: Small and medium enterprises’ perspective

**DOI:** 10.1371/journal.pone.0352724

**Published:** 2026-06-30

**Authors:** The PLOS One Editors

The *PLOS One* Editors issue this Expression of Concern for this article [1] because of concerns identified about the peer review process. Readers are advised to interpret the article [1] with caution. In following up on this matter, PLOS did not evaluate the article’s scientific validity.

The Editors have no evidence of any author involvement in the peer review concerns.
