# Supplementary material for: How innovation funding leads enterprises to engage in research and development: Small and medium enterprises’ perspective
Source: PLoS One. 2023 Jul 25;18(7):e0289166. doi: 10.1371/journal.pone.0289166 (PMC10368280; doi:10.1371/journal.pone.0289166)
Supplement: S1 Data — (ZIP) [file pone.0289166.s001.zip › Data & Description/Description of indicators and data.doc]

**Sample cleaning**

1. We obtained annual data for small and medium-sized enterprises listed on the stock market, covering the period from 2012 to 2019. It is important to note that the annual reports used in this study contain actual recorded information from 2011 to 2018 for each firm.

2. We excluded financial companies, including banks, insurance companies, investment companies, brokerage firms, credit card companies, and accounting firms, from our analysis. Moreover, we removed companies that experienced two consecutive years of losses (ST), as well as those with three or more consecutive years of losses (*ST). Additionally, any companies with invalid data were excluded from the dataset.

3. To address outliers and account for the substantial variation across companies, both government innovation subsidies and R&D investment in SMEs are transformed using the natural logarithm.

4. Given the time lag associated with the signaling mechanism of government subsidies and the need for information asymmetry between enterprises and investors to be fully incorporated, a lag of one year is applied to government innovation subsidies. This lag ensures the reliability and robustness of the theoretical and empirical evidence.

**Data sources**

Enterprise annual reports, CSMAR database (website: <https://www.gtarsc.com/>), RESSET database (website: <http://www.resset.cn/endatabases>).

**Variable construction:**

| Variables | Variable name | Variable label |
| --- | --- | --- |
| **Y** | Firms R&D investment (lnRD) | Total amount of enterprise’s R&D investment (log-transformed) |
| **X** | Government Innovation Subsidies (lnGS) | Total amount of government innovation subsidies the firm gain (log-transformed and lagged values) |
| **Mediator** | Financing constraint (CF) | Segmentation based on the nature of corporate equity, gearing and interest coverage multiples |
|  | Endogenous financing constraints Index (CF1) | Net cash flows from operating activities |
|  | Exogenous Financing Constraints Index (CF2) | Financing constraint indicators constructed using multivariate discriminant analysis for gearing ratio, current ratio, and working capital ratio |
| **Controls** | Business Growth (S) | Operating income growth rate = (ending operating income - opening operating income) / opening operating income |
|  | Corporate profitability (ROE) | Return on Net Assets = Net Income / Net Assets |
|  | Enterprise size (Size) | Natural logarithm of the total assets |
|  | Shareholding Concentration (OC) | Percentage of shareholding of the largest shareholder |
|  | Corporate Investment Opportunities (Q) | Corporate Tobin’s Q |
